# Information Bottleneck as Optimisation Method for SSVEP-Based BCI

**DOI:** 10.3389/fnhum.2021.675091

**Published:** 2021-09-07

**Authors:** Anti Ingel, Raul Vicente

**Affiliations:** Institute of Computer Science, University of Tartu, Tartu, Estonia

**Keywords:** brain-computer interface, steady-state visual evoked potential, information bottleneck, mutual information, optimisation

## Abstract

In this study, the information bottleneck method is proposed as an optimisation method for steady-state visual evoked potential (SSVEP)-based brain-computer interface (BCI). The information bottleneck is an information-theoretic optimisation method for solving problems with a trade-off between preserving meaningful information and compression. Its main practical application in machine learning is in representation learning or feature extraction. In this study, we use the information bottleneck to find optimal classification rule for a BCI. This is a novel application for the information bottleneck. This approach is particularly suitable for BCIs since the information bottleneck optimises the amount of information transferred by the BCI. Steady-state visual evoked potential-based BCIs often classify targets using very simple rules like choosing the class corresponding to the largest feature value. We call this classifier the arg max classifier. It is unlikely that this approach is optimal, and in this study, we propose a classification method specifically designed to optimise the performance measure of BCIs. This approach gives an advantage over standard machine learning methods, which aim to optimise different measures. The performance of the proposed algorithm is tested on two publicly available datasets in offline experiments. We use the standard power spectral density analysis (PSDA) and canonical correlation analysis (CCA) feature extraction methods on one dataset and show that the current approach outperforms most of the related studies on this dataset. On the second dataset, we use the task-related component analysis (TRCA) method and demonstrate that the proposed method outperforms the standard argmax classification rule in terms of information transfer rate when using a small number of classes. To our knowledge, this is the first time the information bottleneck is used in the context of SSVEP-based BCIs. The approach is unique in the sense that optimisation is done over the space of classification functions. It potentially improves the performance of BCIs and makes it easier to calibrate the system for different subjects.

## 1. Introduction

Brain-computer interface (BCI) is a nonmuscular communication channel that can be used, for example, by people with severe motor disabilities to control a computer or another external device. Steady-state visual evoked potential (SSVEP)-based BCIs, in particular, have received much attention over the past few decades due to their ease of use and high information transfer rate (ITR) (Vialatte et al., 2010; Gao et al., 2014). However, the performance in terms of ITR is still considered a key obstacle to real-life applications (Nakanishi et al., 2014, 2018; Chen et al., 2015b). In this study, a method for finding an optimal classification rule for SSVEP-based BCIs is proposed. This method potentially improves the performance of BCIs and makes it easier to calibrate the system for different subjects.

The classification of targets in an SSVEP-based BCI is often done using very simple rules (Cheng et al., 2002; Friman et al., 2007; Lin et al., 2007; Zhang et al., 2014a; Nakanishi et al., 2018; Zerafa et al., 2018; Lee and Choi, 2021), such as checking whether extracted feature exceeds a certain threshold or choosing the target corresponding to the maximum feature value. In this study, we propose using the information bottleneck method (Tishby et al., 1999) to find optimal classification rule automatically. We will also compare the original information bottleneck with the more recently introduced deterministic information bottleneck (Strouse and Schwab, 2017). To our knowledge, the information bottleneck has not been used in the context of SSVEP-based BCIs before.

The information bottleneck introduced by Tishby et al. (1999), is an information-theoretic method for solving a specific optimisation task. In this task, two random variables are considered. The goal is to find a third random variable that is a compressed version of the first variable but still has much information about the second variable. The method aims to find the best trade-off between compressing the representation and preserving meaningful information. The information bottleneck provides a rich framework for discussing problems in signal processing and learning (Tishby et al., 1999), with applications in representation learning or feature extraction in machine learning (Bengio et al., 2013; Shwartz-Ziv and Tishby, 2017; Mukherjee, 2019; Goldfeld and Polyanskiy, 2020; Zaidi et al., 2020). In this study, however, we do not apply the information bottleneck for feature extraction. We use the information bottleneck to find an optimal classification rule. In a broad sense, instead of compressing data into features, we compress features into a classification rule. To our knowledge, the information bottleneck has not been used this way before and, thus, we introduced a new application for the information bottleneck. This approach is particularly suitable for BCIs since the performance measure is related to mutual information (Thompson et al., 2014; Ingel et al., 2018), which is exactly what the information bottleneck maximises.

There are various feature extraction methods available for SSVEP-based BCIs, while classification methods have received less attention. Cheng et al. (2002) introduced the power spectral density analysis (PSDA) method, which extracts features using fast Fourier transform (FFT). Zhang et al. (2010) use the continuous wavelet transform instead of FFT. These methods extract features separately for different channels. Spatial filtering methods allow extracting information from multiple channels; two such methods were introduced by Friman et al. (2007). Lin et al. (2007) introduced the widely used canonical correlation analysis (CCA) method, which also extracts features from multiple channels. The CCA method was later improved by optimising reference signals by Zhang et al. (2011, 2014b). The likelihood ratio test method introduced by Zhang et al. (2014a) achieved slightly better performance than the standard CCA. Nakanishi et al. (2018) introduced the task-related component analysis (TRCA) method to SSVEP-based BCIs, outperforming other existing methods. Lee and Choi (2021) proposed an improved strategy for decoding SSVEPs to overcome some limitations of the TRCA method. Zerafa et al. (2018) give an overview of available methods and compare methods that require training with those that do not. Thus, much research has been done in the context of feature extraction methods, and methods specifically for SSVEP-based BCIs have been introduced.

Classification rules for SSVEP-based BCIs have received less attention. Often classification is done using simple rules including predicting the class corresponding to maximum feature value; we will call this classification rule the argmax classifier. In some studies, standard machine learning methods have been applied for classification. Carvalho et al. (2015) compare linear discriminant analysis (LDA), support vector machine (SVM), and extreme learning machine as classification rules. They obtain the best results with LDA. SVM was also used by Zhang et al. (2010), Anindya et al. (2016), Jukiewicz and Cysewska-Sobusiak (2015), Velchev et al. (2016), while LDA was used by Yehia et al. (2015). Oikonomou et al. (2017) used multiple linear regression under the sparse Bayesian learning framework and showed that their approach outperforms SVM when a small number of channels are used. Du et al. (2020) use a convolutional neural network and show that in terms of accuracy, their approach outperforms some CCA based methods and a previous neural-network-based classifier. Ingel et al. (2018) introduce a classifier specific to BCIs and show that it outperforms a random forest classifier.

A drawback of using standard machine learning methods as a BCI classifier is that these methods are not optimising ITR, which is the primary performance measure for BCIs (Wolpaw et al., 1998; Mowla et al., 2018), but often these methods aim to optimise accuracy. If the classifier is allowed to leave samples unclassified, optimising only the accuracy can lead to unwanted results, such as a very slow but accurate BCI. Allowing the classifier to leave samples unclassified is beneficial because then the classifier can avoid making errors when it is not confident enough in the prediction (Ingel et al., 2018). Using classifiers that optimise mutual information instead of accuracy, as in this study, has benefits also in other contexts (Hu, 2014).

There is a relationship between accuracy and ITR, but if the assumptions of ITR (Wolpaw et al., 1998) are not met, which is often the case in practice (Yuan et al., 2013; Thompson et al., 2014), then ITR can give incorrect estimate of the amount of information transferred by the system (Ingel et al., 2018). How good the estimate is, depends on how seriously the assumptions are violated. For these reasons and following suggestions by Thompson et al. (2014), we use mutual information to estimate the amount of information transferred by the system. The standard ITR is a special case of the mutual-information-based performance measure (Ingel et al., 2018, Appendix E) under the assumptions of ITR (Wolpaw et al., 1998; Thompson et al., 2014).

In this study, we propose a classification method specifically designed for BCIs. It provides more flexibility than the argmax classifier, and unlike standard machine learning methods, it is designed to maximise the amount of information transferred by the BCI. The proposed method uses training on subject-specific data. As shown from the development of feature extraction methods, switching from methods that do not require training to methods that use subject-specific training has improved the performance, refer to review by Zerafa et al. (2018). This study is shown as a step toward similar advancement in classification rules for BCIs.

We evaluate the performance of the proposed method on two publicly available datasets (Bakardjian et al., 2010; Wang et al., 2017). The dataset by Bakardjian et al. (2010) is a smaller dataset containing data for four subjects and three target frequencies. This dataset has been used in several works. From these works, Ingel et al. (2018) report the highest average ITR of 35 bits/min averaged over subjects and ITR of 62 bits/min for Subject 1 while using PSDA and CCA feature extraction and classification rule specifically designed for BCIs. The argmax classifier is used by Demir et al. (2016) (bio-inspired filter banks feature extraction, ITR up to 12 bits/min), Demir et al. (2019) (bio-inspired filter banks feature extraction, classification also uses logistic regression, ITR up to 22.29 bits/min), Karnati et al. (2014) (feature extraction based on fit sine curve amplitudes, accuracies of 0.83–0.92 with window length 0.5 while using two trials of two subjects), Jukiewicz et al. (2018) (CCA feature extraction with blind source separation, ITR up to 18.26 bits/min), Jukiewicz et al. (2019) (modification of CCA feature extraction using genetic algorithms, accuracies around 0.55–0.83 with 1-s window length while using two classes). A threshold-based classifier is used by Saidi et al. (2017); they introduced feature extraction and classification method based on Ramanujan periodicity transform and reported an accuracy of 0.79 with a time window of 1.125 s while using two classes. Standard machine learning methods are used by Velchev et al. (2016) (multiple signal classification feature extraction, SVM classifier, average ITR of 17 bits/min), Jukiewicz and Cysewska-Sobusiak (2015) (PSDA feature extraction, bilinear separation classifier, average ITR of about 10 bits/min), Yehia et al. (2015) (PSDA feature extraction with principal component analysis, LDA classifier, accuracy averaged over subjects and different time windows of 89.5%), Anindya et al. (2016) (PSDA feature extraction, SVM classifier). Thus, most of the works report ITR up to 22 bits/min, while Ingel et al. (2018) achieve an average ITR of 35 bits/min with classification rule specifically designed for BCIs.

The dataset by Wang et al. (2017) is a larger dataset containing data for 35 subjects and 40 target frequencies. Since this dataset has more classes than the dataset by Bakardjian et al. (2010), the achieved ITR is also considerably larger. For example, this dataset has been used by Zhang et al. (2018b), Zhang et al. (2018a), Jiang et al. (2018), Nakanishi et al. (2018), Wong et al. (2020), and they report average ITRs up to 230 bits/min.

Ingel et al. (2018) proposed an algorithm for finding optimal rule from a fixed set of threshold-based classification rules. In particular, they use a threshold-based classification rule which has a threshold for each feature. A sample is classified into a class in their approach if the feature value is above the threshold for this class, and for all the other classes, the feature value is below their corresponding thresholds. If this condition is not met, the sample is left unclassified. They optimise the thresholds by representing ITR as a function of thresholds. Thus choosing the classification rule is reduced to optimising a multivariable function that calculates ITR on the training data given the classification thresholds. This approach seems to outperform other results on the same dataset in terms of ITR, probably because the algorithm directly maximises ITR. We use a similar approach in this study, but we do not have to restrict the approach to threshold-based classifiers thanks to using the information bottleneck. Thus, the set of considered classifiers is much more diverse. Furthermore, Ingel et al. (2018) assume that a larger feature value means that the corresponding class is more likely the correct class. This assumption is not needed in the current approach.

## 2. Methods

### 2.1. Datasets

#### 2.1.1. Dataset 1

The dataset by Bakardjian et al. (2010) contains EEG recordings of four healthy subjects with normal or corrected-to-normal vision. All subjects were naive to the SSVEP-based BCI. There are three targets with frequencies 8, 14, and 28 Hz. The visual stimuli were displayed on a 21” CRT computer monitor with a 170 Hz refresh rate, placed approximately 90 cm away from the eyes of the subject. SSVEP stimulation was achieved using small reversing black and white checkerboards with 6 × 6 checks. For each subject, there are five trials for each target frequency. Each trial consists of 25 s of EEG data, and the first 5 s and last 5 s are without visual stimulation. Dataset was recorded using BIOSEMI EEG (Biosemi Inc., Amsterdam) system with 128 channels and 2,048 Hz sampling rate and downsampled to 256 Hz.

#### 2.1.2. Dataset 2

The dataset by Wang et al. (2017) contains EEG recordings of 35 healthy subjects with normal or corrected-to-normal vision. Eight of the subjects had previous experience of using their SSVEP speller. The remaining 27 subjects were naive to the SSVEP-based BCI. There are 40 targets, and frequencies range from 8 to 15.8 Hz with an interval of 0.2 Hz. The visual stimuli were presented on a 23.6” LCD monitor with a 1920 × 1080 resolution at 60 Hz. The monitor was placed approximately 70 cm away from the subject. SSVEP stimulation was achieved using white squares on black background. Each square had a letter, digit, or some other symbol on it. For each subject, there are six trials for each target frequency. Each trial consists of 6 s of EEG data; the first 0.5 s and the last 0.5 s is without the visual stimulation. Dataset was recorded using Synamps2 EEG system (Neuroscan, Inc.) with 64 channels and 1,000 Hz sampling frequency and downsampled to 250 Hz. Refer to Wang et al. (2017) for more details.

### 2.2. Feature Extraction

#### 2.2.1. Feature Extraction Method for Dataset 1

For feature extraction on Dataset 1, we mainly follow the approach used by Ingel et al. (2018) as they report the highest average ITR on this dataset. The only difference from Ingel et al. (2018) is that we have an additional pre-processing step. In particular, we subtract the Cz channel signal from the O1 and O2 channels as the first step. The rest of the feature extraction is identical to Ingel et al. (2018). In particular, we use channels O1 and O2 from the dataset, and we use PSDA (Cheng et al., 2002) and CCA (Lin et al., 2007) feature extraction methods. For each trial, the first and last 5 s are discarded because there was no visual stimulation. A sliding window with a window length of 1 s is used on the remaining data, and features are extracted after every 0.125 s time step. In the PSDA method, the powers of the first three harmonics and their sum were used as features. In the CCA method, the set of reference signals consisted of standard cosine and sine reference signals for the first three harmonics of the target frequency. Multiple features were combined using an LDA dimensionality reduction. The final features were the distances to LDA decision borders. Refer to Ingel et al. (2018) for more details.

#### 2.2.2. Feature Extraction Method for Dataset 2

For feature extraction on Dataset 2, we follow the approach by Nakanishi et al. (2018) and use the implementation available at a Github repository^1^. In particular, we use data of channels Pz, PO5, PO3, POz, PO4, PO6, O1, Oz, and O2 from the dataset. The first and last 0.5 s of each trial is discarded since there was no visual stimulation. The first 0.134 s of data were discarded from the remaining data since this approximately corresponds to latency delay in the visual pathway. We apply filter bank analysis (Chen et al., 2015a) on the remaining data to decompose EEG data to sub-band components. Then TRCA (Tanaka et al., 2013) is applied, which results in feature values used in classification. Refer to Nakanishi et al. (2018) for more details.

The only difference from Nakanishi et al. (2018) is that we use a sliding window with a window length of 0.5 s in feature extraction and extract features after every 0.125 s time step, similarly as we did for Dataset 1. In this study, we retrain TRCA for every time step. Using a sliding window is required because the proposed algorithm uses feature values as training data, and a single prediction on each trial does not give enough training samples.

### 2.3. Information-Theoretic Quantities

This section gives the definitions of basic information-theoretic quantities needed to introduce the performance measure and the information bottleneck. Let *Y* denote a random variable with a finite number of possible values *y*_1_, …, *y*_*n*_. Then entropy of *Y* is defined as

(1) $$\begin{array}{r} H\left(Y\right)=-\overset{n}{\underset{i=1}{\sum }}\text{P}\left(Y=y_{i}\right)\log_{2}\left(\text{P}\left(Y=y_{i}\right)\right) \end{array}$$

where 0 log_2_ 0 is defined to be equal to 0 and **P** is a probability measure. Let *Z* denote another random variable with a finite number of possible values. The conditional entropy of *Y* given *Z* is defined as

(2) $$\begin{array}{r} H\left(Y|Z\right)=H\left(Y,Z\right)-H\left(Z\right) \end{array}$$

where *H*(*Y, Z*) is the entropy of the random vector (*Y, Z*). Finally, we define mutual information between *Y* and *Z* as

(3) $$\begin{array}{r} I\left(Y;Z\right)=H\left(Y\right)-H\left(Y|Z\right). \end{array}$$

In the following, if *Y* is a random variable and *i* is its possible value, then, we denote the event that the value of *Y* is *i* as *Y*_*i*_.

### 2.4. Information Transfer Rate

A commonly used performance measure for SSVEP-based BCIs is ITR (Wolpaw et al., 1998; Mowla et al., 2018). The number of targets is denoted by *N* and the accuracy of the BCI is denoted by *a*. ITR for a single prediction is defined as given in Equation (4):

(4) $$\begin{array}{r} {\text{ITR}}_{s}=\log_{2}N+a\log_{2}a+\left(1-a\right)\log_{2}\left(\frac{1-a}{N-1}\right). \end{array}$$

This formula is a special case of mutual information between the true target and the predicted target, obtained by assuming that the channel is doubly symmetric (Wolpaw et al., 1998; da Silva Costa et al., 2020). Since the proposed approach does not take into account this assumption and often this assumption is not met in practice (Yuan et al., 2013; Thompson et al., 2014), we use mutual information between the predicted class and correct class as the performance measure as suggested by Thompson et al. (2014).

In more detail, the set of classes is denoted as {1, 2, 3, …, *N*}. The random variables modelling the predicted class and the true class are denoted as *P* and *C*, respectively. The events of the predicted class being *i* and the true class being *j* are denoted as *P*_*i*_ and *C*_*j*_, respectively. Then, *I*(*P*; *C*), as defined in (3), shows the amount of information transferred with a single prediction.

To calculate mean detection time (MDT), we follow the approach of Ingel et al. (2018). In particular, we estimate MDT as follows:

(5) $$\begin{array}{r} \text{MDT}=w+\left(\frac{1}{\text{P}\left({⋃}_{i=1}^{N}P_{i}\right)}-1\right)\cdot s \end{array}$$

where *w* is the window length and *s* is the time step between consecutive feature extractions. In this formula, *s* is multiplied by the expected number of failed classifications before a successful one. Mean detection time is estimated this way due to the overlapping sliding window used in feature extraction.

To get a more accurate estimate of MDT, we would have to include gaze shifting time to MDT, which is considered to be about 0.5 s (Chen et al., 2015b; Nakanishi et al., 2018; Liang et al., 2020). We add this constant for experiments on Dataset 2 but not for experiments on Dataset 1 to have a straightforward comparison to previous results on these datasets. All the probabilities in the previous two formulas are estimated from the confusion matrix of the classifier.

Finally, the performance measure we use is defined as the following:

(6) $$\begin{array}{r} {\text{ITR}}_{mi}=I\left(P;C\right)\frac{60}{\text{MDT}} \end{array}$$

and we also report the standard performance measure defined as

(7) $$\begin{array}{r} \text{ITR}={\text{ITR}}_{s}\frac{60}{\text{MDT}}. \end{array}$$

Both of these are in units of bits per minute.

### 2.5. Discretising Feature Values

Since the information bottleneck algorithm we use requires discrete random variables, we discretise random variables which model the feature values. We explore two different methods for discretising. The first method we consider for discretising the features is simply binning the values. We calculate bins separately for each feature. The random variable modelling unbinned feature value for class *i* is denoted as *F*_*i*_ and binned feature value is denoted as *B*_*i*_. The relationship between *F*_*i*_ and *B*_*i*_ is that if [*l, h*) is one of the bins, then *F*_*i*_ ∈ [*l, h*) happens if and only if *B*_*i*_ = [*l, h*).

After feature extraction on the training dataset, we obtain a collection of realisations of *F*_*i*_ given *C*_*j*_ for all *i, j*. For each *i*, we find the smallest and largest value of *F*_*i*_ and take these as the start of the first bin and end of the last bin, respectively. The number of bins is calculated using different estimators for Dataset 1 and Dataset 2 since Dataset 1 has more samples for each subject. For Dataset 1, we use Freedman Diaconis estimator (Freedman and Diaconis, 1981), which is defined as

(8) $$\begin{array}{r} 2\cdot \frac{\text{IQR}}{\sqrt[{3}]{n}}, \end{array}$$

where *n* is the number of training samples and IQR is the interquartile range. For Dataset 2, we use Sturges' estimator (Sturges, 1926), which is defined as

(9) $$\begin{array}{r} \left\lceil \log_{2}n\right\rceil +1, \end{array}$$

where *n* is again the number of training samples and ⌈·⌉ denotes the ceiling function.

We calculate the number of bins for conditional distributions of *F*_*i*_ given *C*_*j*_ for each class *j* and take the mean of these, rounded to the nearest integer, as the number of bins for feature *F*_*i*_. Then binning feature values to the bins gives a histogram that estimates the probabilities **P**(*B*_*i*_ = *b*_*i*_∣*C*_*k*_) for all *i, k*, and bin *b*_*i*_.

The second method, we consider, for discretising the features is fitting skew-normal distribution to the conditional distributions of *F*_*i*_ given *C*_*j*_ for all *i, j* and calculating the probability from cumulative distribution function, similarly to Ingel et al. (2018). We still calculate the bin edges the same way as in the previous method, but now the probabilities are calculated for a given bin [*l, h*) by using Equation (10):

(10) $$\begin{array}{r} \text{P}\left(B_{i}=\left[l,h\right)|C_{k}\right)=F_{F_{i}|C_{k}}\left(h\right)-F_{F_{i}|C_{k}}\left(l\right) \end{array}$$

where *F*_*F*_*i*_|*C*_*k*__ is the cumulative distribution function of conditional distribution of *F*_*i*_ given *C*_*k*_.

### 2.6. Input to the Information Bottleneck

To use the information bottleneck, we need a single random variable combining all the feature values. Thus, let *X* denote a random variable defined as *X* = (*B*_1_, *B*_2_, …, *B*_*N*_), where *B*_1_, *B*_2_, …, *B*_*N*_ denote the binned feature values as in section 2.5. For any class *k*, we assume that the random variables *B*_1_, *B*_2_, …, *B*_*N*_ are conditionally independent, conditioned on *C*_*k*_. Similar assumption was made by Ingel et al. (2018) for features. This assumption allows us to use the formula

(11) $$\begin{array}{r} \text{P}\left(X=\left(b_{1},b_{2},\ldots ,b_{N}\right)|C_{k}\right)=\overset{N}{\underset{i=1}{\prod }}\text{P}\left(B_{i}=b_{i}|C_{k}\right) \end{array}$$

to calculate input distribution for the information bottleneck. Probabilities on the right hand side are estimated from the histogram or with the skew-normal distribution as discussed in the section 2.5. As before, we denote the event that *X* = *k* for suitable value of *k* as *X*_*k*_.

### 2.7. Information Bottleneck

This section describes the information bottleneck (Tishby et al., 1999) and the deterministic information bottleneck (Strouse and Schwab, 2017). We continue using the notation defined in previous sections. In particular, we use random variables *C* and *P* for true class and predicted class as in section 2.4, and we use *X* as the vector of bins as in section 2.6.

The information bottleneck introduced by Tishby et al. (1999) is an information-theoretic optimisation problem that, given probabilities **P**(*X*_*k*_, *C*_*j*_), finds a solution for the following optimisation problem:

(12) $$\begin{array}{r} \arg\min\left[I\left(P;X\right)-\beta I\left(P;C\right)\right] \end{array}$$

where minimisation is over conditional distributions of *P* given *X*, β is a non-negative parameter that we can choose, and minimisation is done subject to Markov constraint *C* → *X* → *P*, which means that

(13) $$\begin{array}{r} \text{P}\left(P_{i}|C_{j},X_{k}\right)=\text{P}\left(P_{i}|X_{k}\right) \end{array}$$

for all possible values of *i, j, k*. Minimisation over conditional distributions means that we are looking for values **P**(*P*_*i*_∣*X*_*k*_) for all *i, k* that minimise the objective function. In the optimisation problem, we have the mutual information *I*(*P*; *C*), which we want to maximise as this increases the performance measure (6). The term *I*(*P*; *X*) characterises compression.

The deterministic information bottleneck introduced by Strouse and Schwab (2017) is an optimisation problem that differs from the original problem by using a different objective function

(14) $$\begin{array}{r} \arg\min\left[H\left(P\right)-\beta I\left(P;C\right)\right]. \end{array}$$

Term *H*(*P*) characterises the representational cost of *P*.

The deterministic information bottleneck gets its name from the fact that its solution is actually a function (Strouse and Schwab, 2017), meaning that the conditional distribution always has all its probability mass on one value. Thus, it gives a function from feature values to classes and hence can be used as a classifier for the BCI.

Strouse and Schwab (2017) actually introduced a more general notion than deterministic information bottleneck. They used the optimisation problem

(15) $$\begin{array}{r} \arg\min\left[H\left(P\right)-\alpha H\left(P|X\right)-\beta I\left(P;C\right)\right]. \end{array}$$

The optimisation problem (15) is equivalent to original information bottleneck if α = 1. Solution to the deterministic information bottleneck is actually found as a limit of the solutions for (15) in the process α → 0. Values between 0 and 1 interpolate between the original information bottleneck and the deterministic information bottleneck.

### 2.8. Classification Rules

First, the training process is discussed. The input probabilities **P**(*X*_*k*_, *C*_*j*_) for all *k, j* are calculated as discussed in section 2.6. Then, the information bottleneck method outputs the probabilities **P**(*P*_*i*_∣*X*_*k*_) for all *i, k*. However, the information bottleneck algorithm filters out some of the feature values from the input distribution if their probability is close to zero. Samples with these features are left unclassified in the algorithm. Implementation of this algorithm can be found in a Github repository^2^.

The first classifier (Classifier 1) is defined as follows. Recall that the feature vector is a vector of bins, as discussed in section 2.6. The input to the classifier is a feature vector *k*, and the classifier outputs class *i* for which

(16) $$\begin{array}{r} \text{P}\left(P_{i}|X_{k}\right)=1 \end{array}$$

if there exists such a class. If there is no such class, the sample is left unclassified. Not classifying all the samples means that the classifier can achieve higher accuracy at the expense of MDT.

An alternative classification rule that could be used is $\arg\underset{i}{\max}$ **P**(*P*_*i*_ ∣ *X*_*k*_). This rule is equivalent to (16) in the case of deterministic information bottleneck but (16) can leave more samples unclassified when using the original information bottleneck.

**Figure 1:**
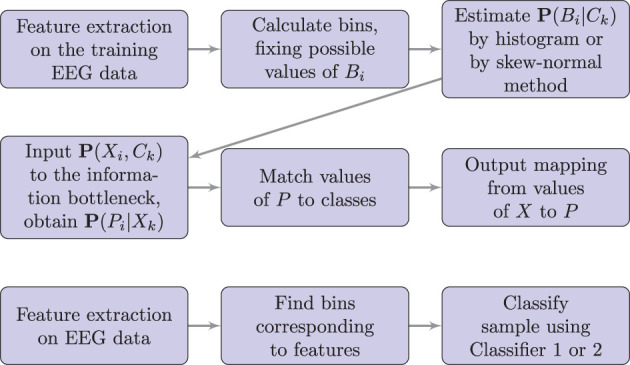
Flowchart of the proposed method. Here *P* denotes the predicted class, *C* denotes the correct class, *B* denotes the bin corresponding to the feature value, and *X* denotes the vector of bins, as defined in sections 2.4–2.6. The upper diagram shows the training process; the lower diagram shows the testing or real-time use.

Also, note that the values of *P* are chosen arbitrarily by the information bottleneck algorithm, and thus, we have to find a mapping from values of *P* to the true classes. We did this by calculating accuracy for each possible mapping and chose the mapping which gives the highest accuracy on the training set. The flowchart of the algorithm is shown in Figure 1.

The idea behind the second classifier (Classifier 2) is that instead of making the classification based on one point in the feature space, the classifier also considers the neighbouring points of the current sample. A prediction is made only if there are enough points classified in the same way among the neighbouring points. Otherwise, the algorithm is considered not confident enough, and no prediction is made. Formally, the bin edges are denoted as $b_{1}^{i},\ldots ,b_{m_{i}}^{i}$ for each feature *i*, and where *m*_*i*_ − 1 is the number of bins for that feature. Assume that these bin edges are in an increasing order and suppose the current sample is corresponded to bins $\left(\left[b_{j_{1}}^{1},b_{j_{1}+1}^{1}\right),\ldots ,\left[b_{j_{N}}^{N},b_{j_{N}+1}^{N}\right)\right)$. Let *n* and *t* denote non-negative integers corresponding to the number of neighbouring points to consider and classification threshold, respectively. Then, the algorithm counts the number of values of *s*_1_, …, *s*_*N*_ ∈ {−*n*, −*n* + 1, …, *n* − 1, *n*} for which the equation

(17) $$\begin{array}{r} \text{P}\left(P_{i}|X=\left(\left[b_{j_{1}+s_{1}}^{1},b_{j_{1}+1+s_{1}}^{1}\right),\ldots ,\left[b_{j_{N}+s_{N}}^{N},b_{j_{N}+1+s_{N}}^{N}\right)\right)\right)=1 \end{array}$$

holds. The number of values of *s*_1_, …, *s*_*N*_ is counted separately for each *i*. If the number of values is larger than *t* for some *i*, then, *i* is taken as the predicted class. Threshold *t* is chosen high enough so that only one class can satisfy the condition.

## 3. Results

To test our approach, we use two publicly available datasets and follow the feature extraction methods by Ingel et al. (2018) and Nakanishi et al. (2018) (details in Methods Section). First, we describe the initial experiments, which motivate the definition of Classifier 2, and motivate the choice of parameters for the following experiments. Then, we evaluate the performance of the proposed classifier on Dataset 1 and Dataset 2.

### 3.1. Initial Experiments

The following analysis is done on Dataset 1. For solving the information bottleneck problems discussed in section 2.7, we used implementation by Strouse and Schwab (2019)^3^. We set the maximum number of values for random variable *P* to 3 as this matched the number of classes in this BCI and ran the algorithm for different values of β. The standard approach to choosing β is to plot *H*(*P*) and *I*(*P*; *C*), in the case α = 0, and plot *I*(*P*; *X*) and *I*(*P*; *C*), in the case α = 1, for different values of β and analyse the plot (Strouse and Schwab, 2019).

In these experiments, we observed that the exact value of β has a very small effect on the solution if it is chosen from an appropriate range. For too small values of β, the algorithm often converges to two or even one value for the random variable *P*, which is undesirable since we want to discriminate between three classes. For very large values of β, the algorithm runs into numerical problems. We calculated the information bottleneck for values of β in {10, 20, 30, …, 150} and observed that the variability of the values of *H*(*P*), *I*(*P*; *C*), and *I*(*P*; *X*) was very small. Results are given in Table 1. For the next experiments, we set β = 100 as having a larger weight on the term appearing in the performance measure (6) was deemed beneficial.

**Table 1:** Information-theoretic quantities for the results of the information bottleneck algorithm.

**Subject**	**α = 0**			**α = 1**		
	***I*(*P*; *C*)**	***H*(*P*)**	***H*(*P*∣*X*)**	***I*(*P*; *C*)**	***I*(*P*; *X*)**	***H*(*P*∣*X*)**
1	0.947 ± 0.000	1.580 ± 0.000	0	0.977 ± 0.000	1.575 ± 0.006	0.004 ± 0.005
2	0.851 ± 0.020	1.551 ± 0.112	0	0.845 ± 0.027	1.511 ± 0.127	0.020 ± 0.027
3	1.069 ± 0.000	1.582 ± 0.000	0	1.069 ± 0.000	1.575 ± 0.008	0.007 ± 0.008
4	0.287 ± 0.015	1.449 ± 0.163	0	0.293 ± 0.003	1.495 ± 0.086	0.063 ± 0.071

*The algorithm was trained using all but the second trial of each subject in Dataset 1. Probabilities are estimated using the skew-normal distribution. Reported values are mean ± SD over β values 10, 20, 30, …, 150 and rounded to three decimal places. Features are the combination of the PSDA and the CCA features. Random variables P, C, and X are the predicted class, the true class, and the feature values (more precisely, the vector of bins), respectively*.

From Table 1, we also see that even if we are using the original information bottleneck, that is α = 1, we still get an almost deterministic solution in most of the cases since the conditional entropy *H*(*P*∣*X*) is very close to zero. Similar results were obtained in case we used only CCA features or only PSDA features. The main difference was that *I*(*X*; *C*) was smaller in these cases (Refer to Table 2).

**Table 2:** Information-theoretic quantities for the results of the information bottleneck algorithm using different features.

**Subject**	**CCA+PSDA**			**CCA**			**PSDA**		
	***I*(*P*; *C*)**		***I*(*X*; *C*)**	***I*(*P*; *C*)**		***I*(*X*; *C*)**	***I*(*P*; *C*)**		***I*(*X*; *C*)**
	**α = 0**	**α = 1**		**α = 0**	**α = 1**		**α = 0**	**α = 1**	
1	0.947 ± 0.000	**0.977 ± 0.000**	1.128	0.921 ± 0.000	0.921 ± 0.000	1.106	0.583 ± 0.000	0.582 ± 0.001	0.760
2	0.851 ± 0.020	0.845 ± 0.027	0.989	**0.869 ± 0.022**	0.864 ± 0.026	0.982	0.637 ± 0.001	0.636 ± 0.002	0.760
3	**1.069 ± 0.000**	**1.069 ± 0.000**	1.187	1.052 ± 0.000	1.051 ± 0.000	1.178	0.762 ± 0.030	0.766 ± 0.029	0.920
4	0.287 ± 0.015	**0.293 ± 0.003**	0.418	0.327 ± 0.021	0.334 ± 0.002	0.396	0.188 ± 0.017	0.196 ± 0.003	0.272

*The algorithm was trained using all but the second trial of each subject in Dataset 1. Probabilities are estimated using the skew-normal distribution. Reported values are mean ± SD over β values 10, 20, 30, …, 150 and rounded to three decimal places. Random variables P, C, and X are the predicted class, the true class, and the feature values (more precisely, the vector of bins), respectively. Bold values correspond to the highest average mutual information between the predicted class and the correct class for each subject*.

**Figure 2:**
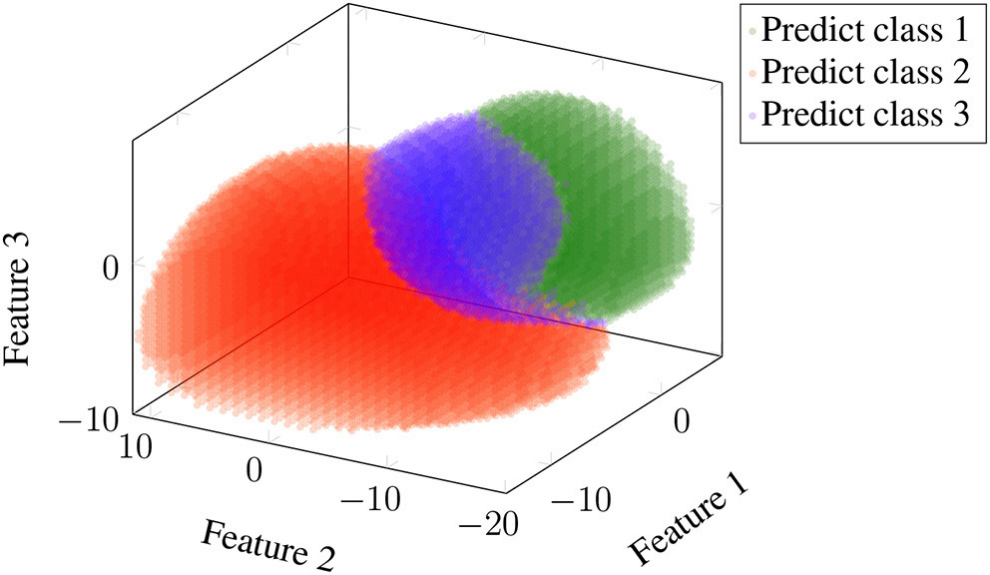
Visualisation of classification results of Classifier 1. This figure shows results for Subject 3 of Dataset 1. All trials but the first were used in training, and features were the combination of canonical correlation analysis (CCA) and power spectral density analysis (PSDA). The information bottleneck parameters were set to α = 1 and β = 100. Probabilities were estimated using the skew-normal distribution. Classification results on training data divide the feature space into three regions based on which the testing data is classified. Classes 1, 2, and 3 correspond to frequencies of 8, 14, and 28 Hz, respectively.

Visualising the classifications made by Classifier 1 on Dataset 1, we observed that if the data is less noisy, for example, for Subject 3, the classifier divides the feature space clearly into three distinct clusters. This provided motivation for the definition of Classifier 2. Refer to Figure 2 for visualisation.

### 3.2. Results on Dataset 1

We evaluated the classification rules introduced in section 2.8 on Dataset 1 using 5-fold cross-validation. In each iteration, four trials were used as a training set, and the remaining trial was used as a test set. In all the experiments, MDT is estimated from the testing data.

For Classifier 2, there are multiple possible values for *n* and *t*. We evaluated Classifier 2 with *n* ∈ {1, 2, 3, 4} and for values of *t* satisfying the Equation (18):

(18) $$\begin{array}{r} {\left(2n+1\right)}^{3}/2<t\leq {\left(2n+1\right)}^{3}. \end{array}$$

**Figure 3:**
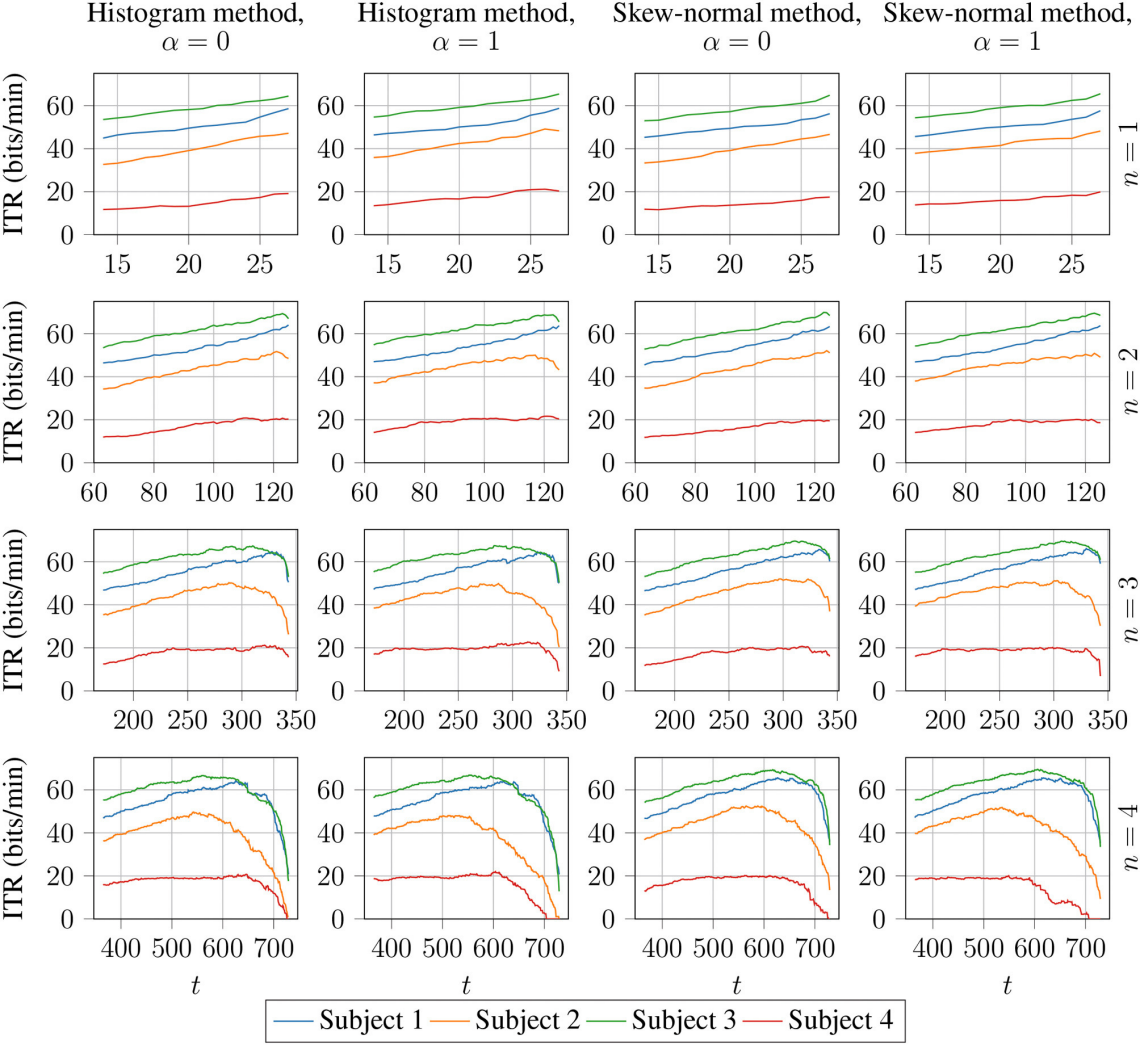
ITR (6) calculated on Dataset 1 for Classifier 2 with *n* ∈ {1, 2, 3, 4} and *t* values as in formula (18). The histogram method and the skew-normal method refer to the methods of estimating probabilities as described in section 2.5.

The lowest value of *t* means that more than half of the neighbouring points have to be classified the same way, and the highest value of *t* means that all of the neighbouring points have to be classified the same way. The results are visualised in Figure 3. This figure shows that there is, in most cases, steady increase in ITR with respect to *t* until a peak value of ITR and then a sudden drop. Thus, the best value of *t* is most likely right before the sudden drop. The best results for Classifier 2 are given in Table 3, where it is shown that it outperforms Classifier 1. Table 4 shows the results for Classifier 2 for *n* = 4 and *t* = 600. As shown, Classifier 2 has higher average ITR compared with the best-performing methods from the related studies.

**Table 3:** ITRs (6) of Classifier 1 and 2 resulting from 5-fold cross-validation.

**Subject**	**Classifier 1**				**Classifier 2**			
	**Histogram**		**Skew-normal**		**Histogram**		**Skew-normal**	
	**α = 0**	**α = 1**	**α = 0**	**α = 1**	**α = 0**	**α = 1**	**α = 0**	**α = 1**
1	45.46	**46.32**	45.19	45.47	64.67	64.64	65.70	**66.13**
2	32.20	34.68	32.66	**36.70**	51.70	50.02	**52.48**	51.82
3	53.54	54.14	53.14	**54.56**	69.35	68.76	**69.91**	69.60
4	11.03	13.74	11.60	**13.95**	21.20	**22.72**	20.68	20.16

*For Classifier 2, these values are the highest ITRs (6) over n and t values. Note that in these results, MDT does not include gaze shift time, and it is estimated from the testing data. Bold values correspond to the highest ITRs (6) of each subject for both classifiers*.

**Table 4:** Classifier 2 compared the related studies.

**Subject**	**Classifier 2**					**Ingel et al. (** **2018** **)**		**Demir et al. (2019)**
	**ITR_*mi*_**	**ITR**	**Accuracy**	**MDT**	**No. pred**.	**ITR_*mi*_**	**ITR**	**ITR**
1	64.84	61.79	0.92	1.09	199	64.62	**62.33**	17.61
2	46.64	**53.48**	0.91	1.22	130	53.73	48.31	17.13
3	69.27	**67.35**	0.94	1.08	212	33.19	27.61	22.29
4	16.42	**16.90**	0.68	1.50	82	5.65	1.74	11.55
Avg	49.29	**49.88**	0.86	1.22	156	39.30	35.00	17.15

*Here n = 4, t = 600, α = 1, and β = 100. The reported values are means resulting from 5-fold cross-validation. Note that in these results, MDT does not include gaze shift time, and it is estimated from testing data. Probabilities are estimated using the skew-normal distribution. No. pred. stands for the number of predictions. Similarly to Classifier 2, Demir et al. (2019) and Ingel et al. (2018) allowed samples to be left unclassified. Bold values correspond to the highest ITR (7) of each subject*.

We performed a paired *t*-test using the average ITR of each subject to check if the differences are statistically significant. The null hypothesis was that the ITR of Classifier 2 is less than or equal to that of Demir et al. (2019), and the alternative hypothesis was that Classifier 2 has higher ITR. We obtained a *p*-value of *p* = 0.02 and a *t*-statistic of 3.51. Therefore, under the assumption that the differences in ITR are normally distributed, the better performance of Classifier 2 is statistically significant. The same comparison between Classifier 2 and the approach of Ingel et al. (2018) did not show statistical significance (*p* = 0.097).

We also evaluated the classifier of Ingel et al. (2018) with the added pre-processing step of subtracting Cz channel from O1 and O2 channels, and we replaced their original gradient descent algorithm with a more stable basin-hopping algorithm (Wales and Doye, 1997). This improved the performance of Subject 3 and Subject 4, giving ITRs similar to the current approach. The obtained ITRs (7) were 60.56, 50.57, 64.08, 21.82 for subjects 1, 2, 3, and 4, respectively. Other related studies mentioned in the introduction either did not report results separately for each subject so that ITR can be obtained, or the reported performance was considerably lower.

### 3.3. Results on Dataset 2

For the experiments on Dataset 2, we set information bottleneck parameters to α = 1, β = 100, and we used the skew-normal distribution method to estimate the required probabilities. We used 6-fold cross-validation to evaluate the performance, each fold corresponding to one trial.

We performed the experiments on subsets of available classes since the exact information bottleneck has high computational complexity in the number of classes. First, we tested the training times of the algorithm in Python programming language on a personal computer with Intel i5-6600 3.30 GHz processor and 8 GB of RAM. Training times for Classifier 2 for 1-fold of the cross-validation were approximately 0.35 s, 7.5 s, 2 min, and 33 min for 2, 3, 4, and 5 classes, respectively. These times were obtained using data of first classes of Subject 1; refer to next paragraph for the order of classes in the dataset. We also evaluated the prediction times using the trained classifier. The prediction times for a single prediction were 60 μs, 100 μs, 1 ms, 75 ms for 2, 3, 4, and 5 classes, respectively. We compared the performance to the argmax classifier, that is, the classifier that predicts the class corresponding to the highest feature value; refer to Nakanishi et al. (2018), for example. The argmax classifier does not require training, and prediction time is around 6 μs for up to 5 classes.

The classes are ordered in the dataset as follows. The first frequencies are from 8 Hz to 15 Hz with a 1 Hz interval. The remaining frequencies are ordered analogically from 8.2 to 15.2, from 8.4 to 15.4, from 8.6 to 15.6, and finally, from 8.8 to 15.8. For simplicity, we refer to the classes according to their order in the dataset. In the following experiments, we calculated the performance measures using consecutive non-overlapping sets of classes. In the case of three classes, we used classes 1, 2, 3, then classes 4, 5, 6, and similarly up to 37, 38, 39, giving 13 different sets of classes in total.

We performed a sign test (Dixon and Mood, 1946) for each set of classes with the null hypothesis that the ITR of Classifier 2 is less than or equal to that of argmax and the alternative hypothesis is that Classifier 2 has a larger ITR. In each case, we obtained a *p*-value less than 0.00002. The results averaged over all the sets of classes is shown in Table 5. An analogical experiment with four classes resulted in a *p*-value less than 0.009 for each set of classes. Thus, the better performance of Classifier 2 is statistically significant. The results averaged over all the sets of four classes is shown in Table 6. In both cases, Classifier 2 has approximately 10 bits/min higher ITR on average.

**Table 5:** Classifier 2 compared with the arg max classifier using three classes.

**Subject**	**Classifier 2**					**argmax classifier**		
	**ITR_*mi*_**	**ITR**	**Acc**	**MDT**	**No. pred**.	**ITR_*mi*_**	**ITR**	**Acc**
1	**90.2**	89.4	0.990	1.02	58	87.2	84.2	0.970
2	**92.2**	92.2	0.997	1.02	59	87.8	85.3	0.974
3	**90.5**	88.7	0.983	1.01	61	86.1	83.5	0.970
4	**91.2**	90.7	0.990	1.01	61	90.1	88.3	0.980
5	92.8	92.9	0.992	1.01	63	**93.5**	92.8	0.995
6	**89.7**	89.3	0.992	1.03	55	83.8	80.7	0.963
7	**88.9**	88.1	0.989	1.03	55	82.4	78.8	0.957
8	**84.6**	83.8	0.982	1.05	47	74.0	69.3	0.927
9	**80.0**	78.6	0.970	1.07	44	65.6	60.5	0.891
10	**86.0**	85.0	0.982	1.04	51	75.5	70.9	0.929
11	**72.5**	69.5	0.948	1.11	37	54.2	48.6	0.839
12	**91.8**	91.6	0.994	1.01	60	87.6	84.9	0.971
13	**87.7**	86.9	0.987	1.03	53	82.3	78.3	0.953
14	**91.8**	91.4	0.993	1.01	61	89.3	87.3	0.980
15	**78.0**	76.2	0.964	1.09	41	64.9	59.7	0.886
16	**73.8**	71.3	0.948	1.09	41	59.0	54.3	0.862
17	**80.4**	79.7	0.971	1.06	45	69.6	63.9	0.904
18	**75.9**	72.8	0.949	1.07	44	60.8	55.4	0.866
19	**41.9**	36.2	0.815	1.30	21	23.9	20.3	0.660
20	**88.3**	87.4	0.989	1.03	53	82.2	79.0	0.958
21	**76.4**	74.5	0.947	1.08	43	66.4	60.8	0.886
22	**93.3**	93.1	0.998	1.01	62	90.2	88.7	0.983
23	**82.9**	81.8	0.971	1.04	51	71.4	66.4	0.908
24	**88.9**	88.7	0.990	1.03	55	82.8	79.1	0.955
25	**91.8**	91.4	0.994	1.01	60	89.4	87.6	0.981
26	**92.1**	91.8	0.994	1.01	61	90.8	89.2	0.983
27	**92.8**	92.6	0.996	1.01	61	90.0	88.2	0.982
28	**89.6**	88.4	0.988	1.02	58	84.6	81.3	0.963
29	**78.1**	75.8	0.964	1.08	42	60.0	55.0	0.868
30	**84.4**	82.7	0.976	1.04	50	76.0	71.5	0.931
31	**94.4**	94.6	1.000	1.00	65	94.2	93.9	0.996
32	**93.7**	93.8	0.999	1.01	62	91.6	90.4	0.989
33	**35.7**	31.0	0.741	1.48	18	28.2	19.5	0.628
34	**91.6**	91.6	0.996	1.02	58	88.7	86.5	0.978
35	**84.7**	84.2	0.985	1.06	46	73.3	69.2	0.923
Avg	**84.0**	82.8	0.970	1.06	52	76.5	72.9	0.925

*Table entries are averages over the sets of three classes of Dataset 2. Here α = 1, β = 100, n = 1, and t = 25, probabilities are estimated with the skew-normal distribution. No. pred. stands for the number of predictions. The number of predictions for the argmax classifier is 66 and MDT is 1 s for each subject. MDT is estimated from test data, and it includes gaze shift time. Bold values correspond to the highest ITR (6) of each subject*.

**Table 6:** Classifier 2 compared with the arg max classifier using four classes.

**Subject**	**Classifier 2**					**argmax classifier**		
	**ITR_*mi*_**	**ITR**	**Acc**	**MDT**	**No. pred**.	**ITR_*mi*_**	**ITR**	**Acc**
1	**113.1**	110.2	0.978	1.02	78	108.8	104.1	0.961
2	**114.4**	111.6	0.981	1.01	80	110.8	106.7	0.969
3	**112.4**	108.1	0.971	1.01	80	107.7	103.3	0.959
4	109.4	105.5	0.957	1.02	79	**113.4**	111.1	0.975
5	**118.1**	117.2	0.994	1.01	84	117.4	116.0	0.992
6	**111.8**	108.5	0.976	1.02	74	105.1	99.9	0.952
7	**109.5**	104.2	0.963	1.02	75	103.3	97.5	0.944
8	**104.2**	100.2	0.964	1.05	63	91.5	83.7	0.902
9	**98.8**	95.4	0.956	1.07	58	81.5	73.3	0.858
10	**106.0**	102.4	0.965	1.04	68	94.9	87.3	0.909
11	**89.1**	82.5	0.919	1.11	50	67.2	59.0	0.798
12	**114.2**	111.7	0.981	1.01	81	110.8	106.5	0.966
13	**108.7**	105.4	0.973	1.04	69	102.5	95.9	0.937
14	**114.4**	110.8	0.976	1.01	81	112.8	109.7	0.976
15	**97.3**	92.5	0.948	1.08	55	82.2	73.5	0.861
16	**92.7**	87.2	0.930	1.08	55	73.3	65.2	0.825
17	**101.2**	97.1	0.955	1.06	61	86.5	77.2	0.871
18	**94.8**	88.1	0.928	1.07	59	77.1	67.9	0.836
19	**50.9**	39.1	0.750	1.29	28	28.5	22.0	0.580
20	**111.6**	109.5	0.984	1.03	73	103.0	97.4	0.945
21	**95.7**	90.9	0.938	1.07	58	82.6	73.1	0.853
22	**116.1**	114.5	0.987	1.01	82	113.6	110.7	0.978
23	**103.0**	98.7	0.955	1.04	67	90.5	82.6	0.889
24	**109.7**	105.3	0.966	1.02	75	104.4	98.6	0.944
25	**113.2**	109.2	0.973	1.02	79	110.6	107.2	0.966
26	**116.0**	114.2	0.987	1.01	82	114.1	111.1	0.978
27	**114.3**	110.1	0.971	1.01	82	113.4	110.4	0.977
28	**111.6**	107.8	0.975	1.02	78	105.8	100.2	0.952
29	**92.7**	87.4	0.934	1.09	55	71.9	64.0	0.820
30	**104.0**	99.3	0.954	1.05	66	94.3	87.1	0.909
31	112.6	110.0	0.967	1.01	84	**119.0**	118.4	0.995
32	**116.1**	113.6	0.983	1.01	83	116.0	114.2	0.987
33	**46.7**	31.8	0.674	1.39	24	37.8	22.0	0.562
34	**112.0**	107.0	0.966	1.02	78	111.5	107.7	0.970
35	**103.1**	98.4	0.956	1.05	63	92.2	85.4	0.905
Avg	**104.0**	99.6	0.950	1.05	69	95.9	90.0	0.906

*Table entries are averages over the sets of four classes of Dataset 2. Here α = 1, β = 100, n = 1, and t = 70, probabilities are estimated with the skew-normal distribution. No. pred. stands for the number of predictions. The number of predictions for the argmax classifier is 88 and MDT is 1 s for each subject. MDT is estimated from test data, and it includes gaze shift time. Bold values correspond to the highest ITR (6) of each subject*.

## 4. Discussion

The results in Table 3 suggest that using the skew-normal distributions to estimate the probabilities gives slightly better results than simply using the histogram. We assume that this is because fitting skew-normal distribution to the data essentially reduces noise in the probability estimates.

Table 3 shows that the original information bottleneck slightly outperforms the deterministic information bottleneck when using Classifier 1. This most likely happens because the original information bottleneck leaves more samples unclassified, in particular, those for which (16) does not hold for any *i*.

Table 2 shows that only the PSDA features and only the CCA features contain less information about the true class (shown by *I*(*X*; *C*)) than their combination. The predicted class can never contain more information about the true class than the features which are used to make the prediction. This is due to the data processing inequality (Cover and Thomas, 2006)

(19) $$\begin{array}{r} I\left(P;C\right)\leq I\left(X;C\right). \end{array}$$

Therefore, using information theory tools, one can evaluate how much information about the true class is lost by different feature extraction methods or in different parts of the BCI pipeline. This can help decide which methods to include in the pipeline without evaluating the final performance.

As shown in section 3.2, the results obtained in this study are similar to the results of Ingel et al. (2018), because we use the same dataset, same feature extraction method, and the classification algorithm in both cases optimises ITR (6). Since two different optimisation methods give similar results, it suggests that these values are close to optimal. However, the current approach has theoretical advantages over Ingel et al. (2018) because fewer assumptions are needed, and optimisation is done over a more diverse set of classifiers.

Tables 3–6 show that for some subjects (Subject 4 in Dataset 1 and Subject 33 in Dataset 2), the performance measures were considerably lower than other subjects. Since this happens with multiple classification methods, we assume that the poor performance is caused by a non-optimal feature extraction method or noisy data for these subjects. This is supported by results in Table 2, which shows that features for Subject 4 contain less information about the true class than for other subjects.

Table 5 shows that Classifier 2 gives better results than the argmax classifier. This is expected since the proposed method finds a classification rule that maximises ITR; thus, it can take into account possible differences between subjects. However, larger ITR can partly be caused by the fact that Classifier 2 can leave samples unclassified when it is not confident enough while the argmax rule classifies all the samples.

With the exact information bottleneck algorithm and low computational power, the method is only applicable in BCIs with a small number of classes as the computational complexity is high in the number of classes. To use this approach with many classes, a more efficient exact information bottleneck algorithm must be used, or the exact information bottleneck algorithm has to be replaced by an approximate one. For an example of an approximate method in deep learning, refer to the approach of Alemi et al. (2017). An overview of currently available information bottleneck approaches is given by Zaidi et al. (2020).

Tables 4–6 show that in some cases, the proposed algorithm is outperformed by the argmax classifier and by the algorithm described by Ingel et al. (2018). We assume this is because the choice of bins was not optimal. Even though the used estimators give bins such that the histogram approximates well the underlying distribution, there might be better choices of bins for the proposed algorithm. Given the best possible choice of bins, the proposed algorithm should outperform the argmax classifier and the algorithm by Ingel et al. (2018) since optimisation is done over a more diverse set of classification rules.

## 5. Conclusion

In this study, using the information bottleneck to find optimal classification rule in SSVEP-based BCIs was proposed. The algorithm was evaluated on publicly available SSVEP datasets. We showed that with the TRCA feature extraction method and a small number of classes, the proposed method outperforms the standard argmax classification rule, achieving 10 bits/min higher average ITR. To use this method with more classes, some approximate information bottleneck algorithm, or a more efficient exact one, must be used. We conclude that using the information bottleneck can make it easier to optimise BCI for different users and improve the performance of the BCI.

## Data Availability Statement

Publicly available datasets were analyzed in this study. This data can be found at: http://www.bakardjian.com/work/ssvep_data_Bakardjian.html, http://bci.med.tsinghua.edu.cn/download.html.

## Author Contributions

AI conceptualised the idea, performed the formal analysis, implemented the algorithm, performed the experiments, and wrote the original draft. RV supervised the study, acquired funding, and reviewed the original draft. Both authors contributed to the article and approved the submitted version.

## Funding

RV thanks the financial support from the EU H2020 program via the TRUST-AI (952060) project. This work was supported by the Estonian Research Foundation via the Estonian Centre of Excellence in IT (EXCITE) (TK148), funded by the European Regional Development Fund.

## Conflict of Interest

The authors declare that the research was conducted in the absence of any commercial or financial relationships that could be construed as a potential conflict of interest.

## Publisher's Note

All claims expressed in this article are solely those of the authors and do not necessarily represent those of their affiliated organizations, or those of the publisher, the editors and the reviewers. Any product that may be evaluated in this article, or claim that may be made by its manufacturer, is not guaranteed or endorsed by the publisher.

## Footnotes

^1^https://github.com/mnakanishi/TRCA-SSVEP (accessed November 8, 2020).

^2^https://github.com/antiingel/information-bottleneck-BCI (accessed November 26, 2020).

^3^https://github.com/djstrouse/information-bottleneck (accessed August 9, 2020).
